# Are There Rearrangement Hotspots in the Human Genome?

**DOI:** 10.1371/journal.pcbi.0030209

**Published:** 2007-11-09

**Authors:** Max A Alekseyev, Pavel A Pevzner

**Affiliations:** Department of Computer Science and Engineering, University of California San Diego, San Diego, California, United States of America; Tübingen University, Germany

## Abstract

In a landmark paper, Nadeau and Taylor [18] formulated the random breakage model (RBM) of chromosome evolution that postulates that there are no rearrangement hotspots in the human genome. In the next two decades, numerous studies with progressively increasing levels of resolution made RBM the de facto theory of chromosome evolution. Despite the fact that RBM had prophetic prediction power, it was recently refuted by Pevzner and Tesler [4], who introduced the fragile breakage model (FBM), postulating that the human genome is a mosaic of solid regions (with low propensity for rearrangements) and fragile regions (rearrangement hotspots). However, the rebuttal of RBM caused a controversy and led to a split among researchers studying genome evolution. In particular, it remains unclear whether some complex rearrangements (e.g., transpositions) can create an appearance of rearrangement hotspots. We contribute to the ongoing debate by analyzing multi-break rearrangements that break a genome into multiple fragments and further glue them together in a new order. In particular, we demonstrate that (1) even if transpositions were a dominant force in mammalian evolution, the arguments in favor of FBM still stand, and (2) the “gene deletion” argument against FBM is flawed.

## Author Summary

Rearrangements are genomic “earthquakes” that change the chromosomal architectures. The fundamental question in molecular evolution is whether there exist “chromosomal faults” (rearrangement hotspots) where rearrangements are happening over and over again. The random breakage model (RBM) postulates that rearrangements are “random,” and thus there are no rearrangement hotspots in mammalian genomes. RBM was proposed by Susumo Ohno in 1970 and later was formalized by Nadeau and Taylor in 1984. It was embraced by biologists from the very beginning due to its prophetic prediction power, and only in 2003 was refuted by Pevzner and Tesler, who suggested an alternative fragile breakage model (FBM) of chromosome evolution. However, the rebuttal of RBM caused a controversy, and in 2004, Sankoff and Trinh gave a rebuttal of the rebuttal of RBM. This led to a split among researchers studying chromosome evolution: while most recent studies support the existence of rearrangement hotspots, others feel that further analysis is needed to resolve the validity of RBM. In this paper, we develop a theory for analyzing complex rearrangements (including transpositions) and demonstrate that even if transpositions were a dominant evolutionary force, there are still rearrangement hotspots in mammalian genomes.

## Introduction

In 1970, Susumu Ohno came up with two fundamental models of chromosome evolution that were subject to many controversies in the last 35 years [1]. One of them (the whole genome duplication model) was first met with skepticism and only recently was proven to be correct [2,3]. The other, the random breakage model (RBM), had a very different fate. It was embraced by biologists from the very beginning (due to its prophetic prediction power) and only recently was refuted by Pevzner and Tesler [4] using a theorem from [5]. However, the rebuttal of RBM caused a controversy and shortly after [4] was published Sankoff and Trinh [6,7] gave a rebuttal of the rebuttal of RBM.

Rearrangements are genomic “earthquakes” that change the chromosomal architectures. The fundamental question in molecular evolution is whether there exist “chromosomal faults” (rearrangement hotspots) where rearrangements are happening over and over again. RBM postulates that rearrangements are “random,” and thus there are no rearrangement hotspots in mammalian genomes.

For the sake of completeness, we give a simple version of both the Pevzner-Tesler and Sankoff-Trinh arguments. Shortly after the human and mouse genomes were sequenced, Pevzner and Tesler [4] argued that if (1) the human–mouse *synteny blocks* are constructed correctly, and (2) chromosomal architectures mainly evolve by the “standard” rearrangement operations (reversals, translocations, fissions, and fusions), then every evolutionary scenario for transforming the mouse genome into the human genome must have a very large number of *breakpoint re-uses*. This result implies that the same regions of the genome are being broken over and over again in the course evolution (rearrangement hotspots), a contradiction to RBM (note that high breakpoint re-use by itself does not invalidate RBM; however, a combination of high breakpoint re-use with scan statistics of the human–mouse breakpoint arrangements invalidates RBM; see Text S1). Consequently, Pevzner and Tesler [4] suggested an alternative fragile breakage model (FBM) of chromosome evolution that was later supported by Murphy et al. [8]. Recent studies further argued for existence of fragile regions (rearrangement hotspots) in mammalian genomes [9–17].

These results are in conflict with the classical Nadeau and Taylor [18] analysis of RBM that implies that there are no rearrangement hotspots in the human genome. In the next two decades, numerous studies with progressively increasing levels of resolution made RBM the de facto theory of chromosome evolution. As a result, the Nadeau-Taylor analysis was until recently viewed as among the most significant results in “... the history and development of the mouse as a research tool” [19]. The paper [4] challenged this view and was quickly followed by other studies questioning the RBM. For example, Kikuta et al. [16] recently wrote “...the results in this study suggest that the Nadeau and Taylor hypothesis is not plausible for the explanation of synteny in general.”

Sankoff and Trinh [6,7] did not question the validity of combinatorial arguments against RBM in [4], but instead argued that the synteny block generation algorithm is parameter-dependent and that question (1) above is more subtle than it may look at first glance. Sankoff and Trinh [6] emphasized how important it is to generate synteny blocks by constructing a series of random rearrangements that create an appearance of breakpoint re-use. They generated a series of random rearrangements according to RBM (i.e., no rearrangement hotspots), computed the resulting synteny blocks, applied the same arguments as in [4], and came to the conclusion that the rearrangement hotspots exist. These hotspots, however, are clearly artifacts of synteny block generation rather than real hotspots, since the simulation in [6] followed RBM.

Recently, Peng et al. [20] re-examined Sankoff and Trinh's arguments and demonstrated that Sankoff and Trinh fell victim to their inaccurate synteny block generation algorithm. Peng et al. [20] further demonstrated that if Sankoff and Trinh had fixed these problems and chosen realistic parameters, their arguments against [4] would disappear. Sankoff recently acknowledged the flaw in [6] (see [21]), and it seems that condition (1) is not controversial anymore. However, Sankoff still appeared reluctant to acknowledge the validity of the Pevzner-Tesler rebuttal of RBM, this time arguing that condition (2) above may also be violated in mammalian evolution. This led to a split among researchers studying chromosome evolution: while most recent studies support the existence of rearrangement hotspots [9–14,16,17], others feel that further analysis is needed to resolve the validity of RBM [22]. Indeed, since the mathematical theory used to refute RBM does not cover more complex rearrangement operations (like transpositions), the arguments in [4] do not apply for the case when transpositions are frequent. In this paper, we develop a theory for analyzing complex rearrangements (including transpositions) and demonstrate that even if transpositions were a dominant evolutionary force, there are still rearrangement hotspots in mammalian evolution. This results in a rebuttal of the rebuttal [21] of the rebuttal [20] of the rebuttal [6,7] of the rebuttal [4] of RBM.

The standard rearrangement operations (i.e., reversals, translocations, fusions, fissions) can be modelled by making two breaks in a genome and gluing the resulting fragments in a new order. One can imagine a hypothetical *k*-break rearrangement operation that makes *k* breaks in a genome and further glues the resulting pieces in a new order. In particular, the human genome can be modelled as the mouse genome broken into ≈280 pieces that are glued together in the “mouse” order. Sankoff [21] is correct in stating that the rebuttal of RBM is not applicable if there was a significant presence of *k*-break rearrangements for large *k* (in fact, it was acknowledged in [4]). However, rearrangements are rare evolutionary events and, starting from the classical Dobzhansky and Sturtevant studies of *Drosophila,* most biologists believe that *k*-break rearrangements are unlikely for *k* > 3, and relatively rare for *k* = 3 (at least in mammalian evolution). Indeed, biophysical limitations and selective constraints are already severe for *k* = 2, let alone for *k* > 2. However, 3-break rearrangements (e.g., transpositions) undoubtedly happen in evolution, although it is still unclear how frequent they are in mammalian evolution. Also, in radiation biology, chromosome aberrations for *k* > 2 (indicative of chromosome damage rather than evolutionary viable variations) may be more common (e.g., complex rearrangements in irradiated human lymphocytes [23–26]). Thus, both the existing controversy about RBM and radiation/cancer biology call for studies of *k*-break rearrangements for *k* > 2.

We recently proved the duality theorem for the *k*-break distance between multichromosomal genomes, and showed how to compute it [27]. In this paper, we focus on the case *k* = 3 (the most relevant case in evolutionary studies) and show that even if 3-break rearrangements were frequent, the Pevzner-Tesler argument against RBM still stands. We further discuss the claim [7,21] that deletion of short synteny blocks may also create an appearance of high breakpoint re-use, an argument against FBM. We invalidate this argument by showing that deletion of short blocks does not lead to increase in breakpoint re-use under the realistic choice of parameters.

## Results

### Multi-break Rearrangements and Breakpoint Graphs

**Figure 1:**
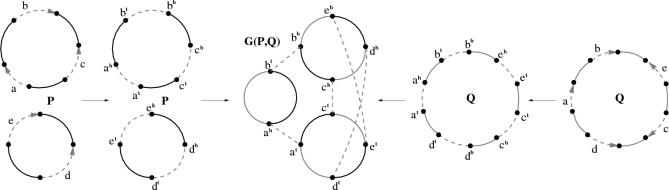
The Breakpoint Graph The breakpoint graph *G*(*P*,*Q*) of a two-chromosomal genome *P* = (+ *a* + *b* − *c*) (−*d* + *e*) and a unichromosomal genome *Q* = (+*a* + *b* – *e* + *c* − *d*) represented as two black-obverse cycles and a gray-obverse cycle, correspondingly.

We start our analysis with *circular genomes* (i.e., genomes consisting of circular chromosomes). We will find it convenient to represent a circular chromosome with genes *x_1_,...,x_n_* as a cycle (Figure 1) composed of *n* directed labeled edges (corresponding to genes) and *n* undirected unlabeled edges (connecting adjacent genes). The directions of the edges correspond to *signs* (strand) of the genes. We label the *tail* and *head* of a directed edge *x_i_* as *x_i_^t^* and *x_i_^h^*, respectively. Vertex *x_i_^t^* is called the *obverse* of vertex *x_i_^h^*, and vice versa. Vertices in a chromosome connected by an undirected edge are called *adjacent.* We represent a genome as a graph consisting of disjoint cycles (one for each chromosome). The edges in each cycle alternate between two colors: one color (usually black or gray) is reserved for undirected edges, and the other color (traditionally called “obverse” and portrayed by dashed lines in Figure 1) is reserved for directed edges. We do not explicitly show the directions of obverse edges since they are defined by superscripts “*t*” and “*h*” (Figure 1).

**Figure 2:**
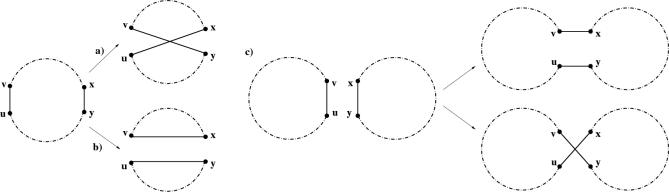
Different Types of 2-Breaks A 2-break on edges (*u*,*v*) and (*x*,*y*) corresponding to (A) reversal: the edges belong to the same black-obverse cycle that is rearranged after 2-break; (B) fission: the edges belong to the same black-obverse cycle that is split by 2-break; and (C) translocation/fusion: the edges belong to different black-obverse cycles that are joined by 2-break.

Let *P* be a genome represented as a collection of *alternating* black-obverse cycles (a cycle is alternating if the colors of its edges alternate). For any two black edges (*u*,*v*) and (*x*,*y*) in the genome (graph) *P*, we define a *2-break* rearrangement as replacement of these edges with either a pair of edges (*u*,*x*), (*v*,*y*), or a pair of edges (*u*,*y*), (*v*,*x*) (Figure 2). 2-Breaks correspond to standard rearrangement operations of reversals (Figure 2A), fissions (Figure 2B), or fusions/translocations (Figure 2C). This definition of elementary rearrangement operations follows the standard definitions of reversals, translocations, fissions, and fusions for the case of circular chromosomes. For circular chromosomes, fusions and translocations are not distinguishable; i.e., every fusion of circular chromosomes can be viewed as a translocation and vice versa. The 2-break rearrangements can be generalized as follows. Given *k* black edges forming a *matching* (i.e., a vertex-disjoint set of edges) on 2*k* vertices, define a *k*-break as replacement of these edges with a set of *k* black edges forming another matching on the same set of 2*k* vertices. Note that a 2-break is a particular case of a 3-break (as well as of a *k*-break for *k* > 3), in which case only two edges are replaced and the third one remains the same.

Let *P* and *Q* be two signed genomes on the same set of genes 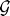 . The *breakpoint graph G*(*P*,*Q*) is defined on the set of vertices *V* = {*x^t^,x^h^* | *x* ∈ 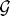 } with edges of three colors: obverse, black, and gray (Figure 1). Edges of each color form a matching on *V: obverse matching* (pairs of obverse vertices), *black matching* (adjacent vertices in *P*), and *gray matching* (adjacent vertices in *Q*). Every pair of matchings forms a collection of alternating cycles in *G*(*P*,*Q*) called *black-gray*, *black-obverse*, and *gray-obverse* cycles, respectively. The chromosomes of the genome *P* (respectively, *Q*) can be read along black-obverse (respectively, gray-obverse) cycles. The black-gray cycles in the breakpoint graph play an important role in analyzing rearrangements [28] (see Chapter 10 of [29] for background information on genome rearrangements).

### Multi-Break Distance between Circular Genomes

The *k*-break distance *d_k_*(*P,Q*) between circular genomes *P* and *Q* is defined as the minimum number of *k*-breaks required to transform one genome into the other. Every *k*-break in the genome *P* corresponds to a transformation of the breakpoint graph *G*(*P*,*Q*). Since the breakpoint graph of two identical genomes is a collection of *trivial* black-gray cycles with one black and one gray edges (the identity breakpoint graph), the problem of transforming the genome *P* into the genome *Q* by *k*-breaks can be formulated as the problem of transforming the breakpoint graph *G*(*P*,*Q*) into the identity breakpoint graph *G*(*Q*,*Q*).

Different from the genomic distance problem [5,30,31] (for linear multichromosomal genomes), the 2-break distance problem for circular multichromosomal genomes has a trivial solution (first given in [32] in a slightly different context). For the sake of completeness, we reproduce a proof from [33]:

**Theorem 1.** *The 2-break distance between circular genomes P and Q is* |*P*| − *c(P,Q) where c(P,Q) is the number of black-gray cycles in G(P,Q).*

**Proof.** It is easy to see that every nontrivial black-gray cycle in the breakpoint graph *G*(*P,Q*) can be split into two by a 2-break, implying that *d_2_*(*P*,*Q*) ≤ |*P*| − *c*(*P*,*Q*). Since every 2-break adds two new edges, it can create at most two new black-gray cycles. On the other hand, since every 2-break removes two old edges, it should remove at least one old black-gray cycle. Hence, no 2-break can increase the number of black-gray cycles by more than one, implying that *d_2_*(*P*,*Q*) ≥ |*P*| − *c*(*P*,*Q*). Therefore, *d_2_*(*P*,*Q*) = |*P*| − *c*(*P*,*Q*). Q.E.D.

**Figure 3:**
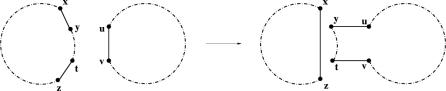
Example of a 3-Break That Corresponds to a Transposition A 3-break on edges (*u*,*v*), (*x*,*y*) and (*z*,*t*) corresponding to a transposition of the segment *y…t* from one chromosome to another. A transposition cuts off a segment of one chromosome and inserts it into the same or another chromosome. A transposition of a segment *π_i_π_i+1_…π_j_* of a chromosome *π_1_…π_i-1_π_i_π_i+1_…π_j_π_j+1_…π_k-1_π_k_…π_n_* into a position *k* of the same chromosome results in a chromosome *π_1_…π_i-1_π_j+1_…π_k-1_π_i_π_i+1_…π_j_π_κ_…π_n_*. For chromosomes *π* = *π_1_…π_m_* and *σ* = *σ_1_…σ_n_*, a transposition of a segment *π_i_π_i+1_…π_j_* of chromosome *π* into a position *k* in the chromosome *σ* results in chromosomes *π_1_…π_i-1_π_j+1_π_j+2_…π_m_* and *σ_1_…σ_k-1_π_i_π_i+1_…π_j_σ_k_…σ._n_*. Underlining shows a piece of chromosome that was transposed from one chromosome to another.

While 2-breaks correspond to standard rearrangements, 3-breaks add transposition-like operations (transpositions and inverted transpositions) as well as three-way fissions to the set of rearrangements (Figure 3). Different from standard rearrangements (modeled as 2-breaks), transpositions introduce three breaks in the genome, making them notoriously difficult to analyze. Computing the minimum number of transpositions transforming one genome into another is called “sorting by transpositions.” A number of researchers considered transpositions in conjunction with other rearrangement operations [34–40]. Despite many studies, the complexity of sorting by transpositions remains unknown [41–45].

Let *c^odd^*(*P*,*Q*) be the number of black-gray cycles in the breakpoint graph *G*(*P*,*Q*) with an odd number of black edges (*odd cycles*).

**Theorem 2.** *The 3-break distance between circular genomes P and Q is* (|*P*| − *c^odd^*(*P*,*Q*)) / 2.

**Proof.** It is easy to see that as soon as there is a nontrivial black-gray odd cycle in the breakpoint graph *G*(*P*,*Q*), it can be split into three odd cycles by a 3-break, thus increasing the number of odd cycles by two. On the other hand, if there exists a black-gray even cycle, it can be split into two odd cycles, thus again increasing the number of odd cycles by two. Therefore, there exists a series of (|*P*| − *c^odd^*(*P*,*Q*)) / 2 3-breaks transforming *G*(*P*,*Q*) into the identity breakpoint graph, implying that *d_3_*(*P*,*Q*) ≤ (|*P*| − *c^odd^*(*P*,*Q*)) / 2. On the other hand, since no 3-break can increase the number of black-gray cycles by more than two, we have *d_3_*(*P*,*Q*) ≥ (|*P*| − *c^odd^*(*P*,*Q*)) / 2. Therefore, *d_3_*(*P*,*Q*) = (|*P*| − *c^odd^*(*P*,*Q*)) / 2. Q.E.D.

For the sake of completeness, below we formulate the duality theorem for the *k*-break distance for an arbitrary *k* from [27]. A subset of cycles in the breakpoint graph *G*(*P*,*Q*) is called *breakable* if the total number of black edges in these cycles equals 1 modulo (*k* − 1). Let *s_k_*(*P,Q*) be the maximum number of disjoint breakable subsets in *G*(*P*,*Q*). For example, for *k* = 3, every odd cycle forms a breakable subset and every breakable subset must contain at least one odd cycle, implying that *s_3_*(*P,Q*) = *c^odd^*(*P,Q*).

**Theorem 3.** *The k-break distance between circular genomes P and Q is* 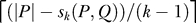 .

### Transpositions and breakpoint re-use.

Sankoff summarized arguments against FBM in the following sentence [21]:

“...And we cannot infer whether mutually randomized synteny block orderings derived from two divergent genomes were created through bona fide breakpoint re-use or rather through noise introduced in block construction or through processes other than reversals and translocations.”

Below we consider the “other processes” argument. The “noise in block construction” argument consists of two parts: synteny block generation and gene deletion. The flaw in the first argument was revealed in [20]. The second argument (“gene deletion”) is analyzed after the “other processes” argument.

In this paper, we study transformations between the human genome *H* and the mouse genome *M* with 3-breaks, using the 281 synteny blocks from [46] and assume that all chromosomes are circular. While analyzing linear chromosomes would be more adequate than analyzing their circularized versions, it poses additional algorithmic challenges that remain beyond the scope of this paper. The related paper [47] addressed these challenges and demonstrated that switching from linear to circular chromosomes does not lead to significant changes in the multi-break distance.

The breakpoint graph *G*(*H*,*M*) contains 35 black-gray cycles, including three odd black-gray cycles, implying that *d_2_*(*H,M*) = 246 (Theorem 1) and *d_3_*(*H,M*) = 139 (Theorem 2). If each of 139 3-breaks on a shortest evolutionary path from *H* to *M* made three breaks, it would imply that there were 139 × 3 – 281 = 136 breakpoint re-uses (for this particular evolutionary path), resulting in the breakpoint re-use rate 1.48 (see Peng et al. [20]). While this is a high breakpoint re-use rate (inconsistent with RBM and the scan statistics), this estimate relies on the assumption that each 3-break on the evolutionary path from *H* to *M* makes three breaks (*complete* 3-breaks). In reality, some 3-breaks can make two breaks (*incomplete* 3-breaks) as 2-breaks are particular cases of 3-breaks, reducing the estimate for the number of breakpoint re-uses. Moreover, the minimum number of breakpoint re-uses may be achieved on a suboptimal evolutionary path from *H* to *M.*

The rebuttal of RBM raises a question about finding a transformation of *H* into *M* by 3-breaks that makes the minimal number of individual breaks. The following theorem shows that there exists a series of 3-breaks that makes the minimum number of breaks while transforming *P* into *Q.*

**Theorem 4.** *Any series of m k-breaks transforming a circular genome P into a circular genome Q makes at least m + d_2_(P,Q) breaks. Moreover, there exists a series of d_3_(P,Q) 3-breaks transforming P into Q that makes d_3_(P,Q) + d_2_(P,Q) breaks.*

**Proof.** For each *k*-break operation, let Δ(*cycles*) be the increase in the number of cycles and Δ(*breaks*) be the increase in the number of breaks. It is easy to see that Δ(*cycles*) ≤ *Δ*(*breaks*) − 1. Summing up over a series of *m k*-breaks transforming *P* into *Q*, we have *|P|* − *c*(*P,Q*) ≤ *b* − *m*, where *b* is the total number of breaks made in the series. Therefore, *b* ≥ |*P*| − *c*(*P,Q*) + *m* = *d_2_*(*P,Q*) + *m*.

Consider a shortest series of complete 3-breaks transforming every odd black-gray cycle into a trivial cycle and every even black-gray cycle into trivial cycles and a single cycle with two black edges. This series consists of *d_3_*(*P,Q*) − *c^even^*(*P,Q*) 3-breaks and results in *c^even^*(*P,Q*) cycles with two black edges that can be transformed into trivial cycles with a series of *c^even^*(*P,Q*) incomplete 3-breaks (i.e., 2-breaks). The total number of 3-breaks in this transformation is *d_3_*(*P,Q*), and they make *3*(*d_3_*(*P,Q*) − *c^even^*(*P,Q*)) + *2c^even^*(*P,Q*) = *3d_3_*(*P,Q*) − *c^even^*(*P,Q*) = *d_3_*(*P,Q*) + *d_2_*(*P,Q*) breaks overall. Q.E.D.

**Corollary 5.** *Every transformation between the circularized human genome H and mouse genome M by 3-breaks requires at least 104 breakpoint re-uses (implying that there exist rearrangement hotspots in the human genome).*

**Proof.** Any transformation of *H* into *M* requires at least *d_3_*(*H,M*) + *d_2_*(*H,M*) = 139 + 246 = 385 breaks. Since there are 281 breakpoints between the human and mouse genomes, it implies that there were at least 385 − 281 = 104 breakpoint re-uses on the evolutionary path from human to mouse, resulting in breakpoint re-use rate 1.37. This is still higher than the expected breakpoint re-use rate of RBM as computed by scan statistics (see [4] and simulations in the next section). It provides an argument against RBM not only for *k* = 2 but also for *k* = 3 and invalidates arguments from [21] in the case *k* = 3 (see also [47]). Since *k*-breaks for *k* > 3 were never reported in previous evolutionary studies, it is unlikely that they significantly affect our conclusions. Q.E.D.

Theorem 4 implies that any transformation of the human genome *H* into the mouse genome *M* with 2-breaks makes at least *d_2_*(*H,M*) *+ d_2_*(*H,M*) = 246 + 246 = 492 breaks, while any transformation of *H* into *M* with 3-breaks makes at least *d_3_*(*H,M*) *+ d_2_*(*H,M*) = 139 + 246 = 385 breaks. Below, we show how the number of breaks made in a series of 3-breaks depends on the number of complete 3-breaks in this series.

**Theorem 6.** *For any series of m 3-breaks transforming a genome P into a genome Q with t complete 3-breaks, m* ≥ *max{d_2_(P,Q)* − *t, d_3_(P,Q)}. Moreover, there exists a series of max{d_2_(P,Q)* − *t, d_3_(P,Q)} 3-breaks transforming P into Q with at most t complete 3-breaks.*

**Proof.** Since *k*-break can increase the number of cycles in the breakpoint graph by at most *k* − 1, a series with *t* complete 3-breaks and *m* − *t* incomplete 3-breaks (i.e., 2-breaks) can increase the number of cycles by at most *2t* + (*m* − *t*) = *m* + *t*. If it transforms the genome *P* into the genome *Q*, then *m* + *t* ≥ *|P|* − *c*(*P,Q*) = *d_2_*(*P,Q*). Therefore, *m* ≥ *d_2_*(*P,Q*) − *t*.

Consider a series of complete 3-breaks, transforming every black-gray cycle with *q* ≥ 3 black edges into two trivial cycles and a cycle with *q* − 2 black edges. Note that such a series may have at most *d_3_*(*P,Q*) − *c^even^*(*P,Q*) 3-breaks (the longest possible series results in *c^even^*(*P,Q*) cycles with two black edges and *|P|* − *c^even^*(*P,Q*) trivial cycles). Since every such 3-break increases the number of cycles by two, a series of *q* = *min* {*t, d_3_*(*P,Q*) − *c^even^*(*P,Q*)} such 3-breaks results in *c*(*P,Q*) + *2q* cycles. These cycles can be transformed into trivial cycles with a series of *|P|* − (*c*(*P,Q*) + *2q*) = *d_2_*(*P,Q*) − *2q* 2-breaks. The total number of 3-breaks and 2-breaks in this transformation is *q* + *d_2_*(*P,Q*) − *2q* = *d_2_*(*P,Q*) − *min* {*t, d_3_*(*P,Q*) − *c^even^*(*P,Q*)} = *max* {*d_2_*(*P,Q*) − *t, d_3_*(*P,Q*)}. Q.E.D.

Theorems 4 and 6 imply:

**Corollary 7.** *Any series of 3-breaks with t complete 3-breaks, transforming a genome P into a genome Q, makes at least d_2_(P,Q)* + *max {d_2_(P,Q)* − *t, d_3_(P,Q)} breaks. In particular, any such series of 3-breaks with t ≤ d_2_(P,Q)* − *d_3_(P,Q) complete 3-breaks makes at least 2d_2_(P,Q)* − *t breaks.*

**Figure 4:**
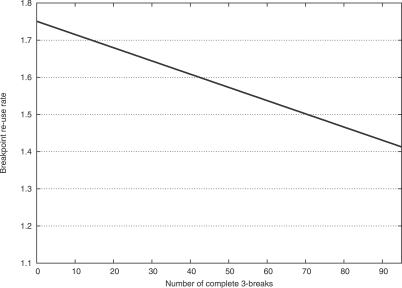
Breakpoint Re-Use Rate as a Function of the Number of Complete 3-Breaks A lower bound for the breakpoint re-use rate as a function of the number of complete 3-breaks in a series of 3-breaks between the circularized human and mouse genomes based on 281 conserved segments from [46]. In the case of linear genomes, the plot is similar, with the breakpoint re-use rate of ≈0.1 lower than in the circular case [47]. In particular, even in the extreme case when the number of transpositions is not limited, the breakpoint re-use rate of ≈1.31 is still higher than the breakpoint re-use rate expected for RBM (see [4]).

Corollary 7 gives the lower bound for the breakpoint re-use rate as a function of the number of complete 3-breaks (i.e., transpositions and three-way fissions) in a series of 3-breaks transforming one genome into the other. For the human genome *H* and mouse genome *M,* this lower bound is shown in Figure 4.

Corollaries 5 and 7 address only the case of circularized chromosomes and further analysis is needed to extend it to the case of linear chromosomes (see [47]). Recently, Bergeron et al. [48] described another promising approach to analyzing both circular and linear chromosomes (using double-cut-and-join operations proposed in [32]) that also opens a possibility to obtain the breakpoint re-use estimates for linear genomes. However, the above estimate is based on the extreme assumption that certain 3-breaks (transpositions and three-way fissions/fusions) represent the dominant rearrangements while reversals and translocations are extremely rare (contrary to the existing view). We emphasize that we do not share the point of view that genomes mainly evolve by transpositions and three-way fissions/fusions, and that we analyzed this assumption only to refute the arguments against FBM. A more realistic analysis of 3-breaks leads to a much higher estimate of the breakpoint re-use (see Figure 4).

### Deletion of Short Blocks and Breakpoint Re-Use

The papers [7,21] claim that deletion of some synteny blocks in [4] may create an appearance of breakpoint re-use even if there was no breakpoint re-use at all. Below, we show that this argument suffers from the same problem (unrealistic parameter choice) that was revealed in [20]. Sankoff and Trinh acknowledged the problem with unrealistic parameter choice in [6] in application to synteny block generation:

“...In fact, Pavel Pevzner (personal communication) has pointed out likely errors in our simulation procedure. Subsequent experiments showed that with realistic sizes and numbers of short inversions, unrealistically large number of long inversions were necessary for the amalgamation process to have an effect...”

Despite the importance of choosing realistic parameters, the paper [7] has no discussion of parameters that are relevant to the human–mouse analysis. Below, we study the deletion process, reproduce simulations in [7], and show that if Sankoff and Trinh used realistic parameters they would confirm (rather than refute) FBM.

**Figure 5:**
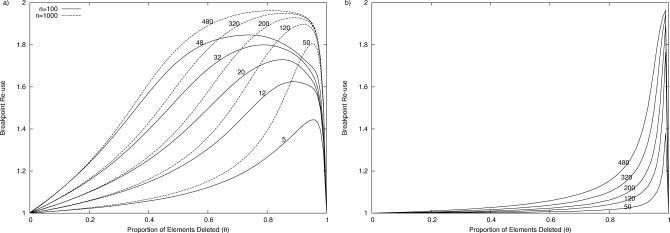
Breakpoint Re-Use Rate as a Function of *θ*, the Proportion of the Elements Deleted (A) Breakpoint re-use rate for parameters *n* = 100 (*m* = 5, 12, 20, 32, and 48) and *n* = 1,000 (*m* = 50, 120, 200, 320, and 480), where *n* stands for the number of elements (genes) and *m* stands for the number of reversals. Since we reproduced simulations in [7], this figure and Figure 1 from [7] are identical. Detailed description (including pseudocode) of this simulation is given in [20]. (B) Breakpoint re-use rate for parameters *n* = 25,000 (*m* = 50, 120, 200, 320, and 480).

Sankoff and Trinh [7] show that deletion of a large number of elements (genes) from a permutation produced by “random” rearrangements would produce a permutation with large breakpoint re-use (Figure 5A). This is not surprising—the only question is what is the realistic number of deleted elements (we use the term “deleted elements” instead of the term “deleted blocks” in [7] to avoid confusion with synteny blocks) to match the reality of human–mouse comparison. If this number does not match the reality of human–mouse comparison, then the observation that the breakpoint re-use increases with element deletions turns into a purely mathematical statement that we are not debating and that is irrelevant to the conclusion in [4] about breakpoint re-use in *mammalian* evolution. For example, if only 20% of all elements are deleted, then Figure 5A (reproduced from [7]) supports rather than rejects FBM (low breakpoint re-use at *θ* = 0.2). However, if one deletes 50% of all elements, the breakpoint re-use becomes rather high, and the Sankoff-Trinh argument against [4] stands. This observation seems to imply that a long-standing debate must be easy to resolve—one should compute the number of deleted elements (genes?) in the human genome and consult Figure 5A. Unfortunately, since it is unclear how one can estimate the number of deleted elements, Figure 5A cannot refute or validate FBM.

**Figure 6:**
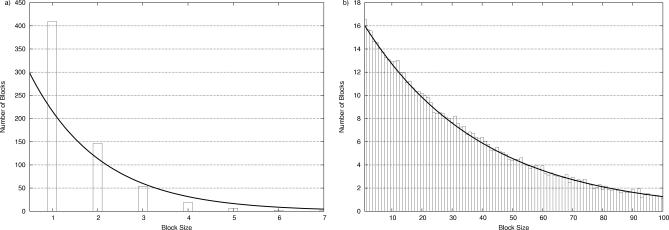
Distribution of Synteny Block Sizes (A) Synteny block sizes (for a permutation with 1,000 elements after 320 reversals) do not fit the exponential distribution expected from RBM. (B) Synteny block sizes (for a permutation with 25,000 elements after 320 reversals) fit the exponential distribution expected from RBM.

The inability to connect Figure 5A with the realities of human–mouse genomic architectures is only part of the problem with the simulations in [7]. Another problem is the parameter choice; for example, it is not clear why the parameter *n* = 100 in Figure 5A is chosen, since the number of rearrangements between the human and mouse genomes clearly exceeds 100. Moreover, most plots for *n* = 1,000 in Figure 5A (particularly those with high breakpoint re-use) produce synteny blocks that do not even fit RBM, which [7] is arguing for. Figure 6A shows that the distribution of synteny block sizes (for *n* = 1,000 elements and *m* = 320 rearrangements, averaged over 100 simulations) is quite different from the exponential distribution characteristic for RBM. In fact, ≈400 out of 640 resulting synteny blocks have (minimal) size 1 (compare with Figure 1, middle panel, in [4] that is used as an argument against RBM). One can argue that Sankoff and Trinh [7] are only interested in reversal distance of the resulting synteny block arrangements, and the sizes of the synteny blocks do not matter. While this argument is correct for *θ* = 0, it becomes flawed for *θ* > 0, since the results of the deletion process are highly dependent on the distribution of the synteny block sizes. Short synteny blocks (of size 1) are “easy” to delete and the unrealistically high proportion of such blocks in the Sankoff-Trinh simulation makes the plot in Figure 5A look quite different from what one would expect if the simulations would follow RBM.

This particular deficiency of the Sankoff-Trinh simulations is easy to fix: one should simply increase the granularity (i.e., increase *n*) to better model RBM. Figure 5B shows the results of simulations with *n* = 25,000 (rough estimate of the number of genes in mammalian genomes) and *m* = 320, while Figure 6B shows that the distribution of the sizes of the synteny blocks (for these parameters) fits the exponential curve and is consistent with RBM). If Sankoff and Trinh presented a (more realistic) plot in Figure 5B in their paper, they would likely confirm rather than refute FBM—indeed, one needs to delete more than 90% of genes (elements) to see significant breakpoint re-use. The sequenced mammalian genomes do not show any evidence of such extreme gene loss. However, although the plot in Figure 5B shows small breakpoint re-use (for any realistic choice of parameters), we prefer not to use it as a counter-argument against the Sankoff-Trinh argument since (similarly to [7]) we do not know what is the best way to choose the parameters (e.g., the number of rearrangements) matching the realities of the human–mouse analysis.

This problem did not escape the attention of Pevzner and Tesler [4]; in fact, they implicitly constructed an analog of Figure 5 and even described the scan statistics to analyze it. The only difference is that instead of parameter *θ* (the number of deleted blocks), they used different parameters *σ* (the minimum size of a synteny block, all smaller ones are deleted) and *γ* (the total size of deleted synteny blocks). Although all these parameters seem to be interchangeable, there is a big difference between them when it comes to the real human–mouse comparison: *σ* and *γ*, different from *θ*, are easy to estimate. Indeed, the key conclusion of [4] is that the large synteny blocks (>1 Mb) cover almost the entire genome (95%), while breakpoint regions (where elusive short synteny blocks hide) cover only ≈5% of the human genome. We emphasize that synteny blocks in [4] are hardly controversial since all follow-up studies with different synteny block generation algorithms came up with roughly the same set of blocks. These blocks are further confirmed by a large number of genes (in the same conserved order with few micro-rearrangements).

**Figure 7:**
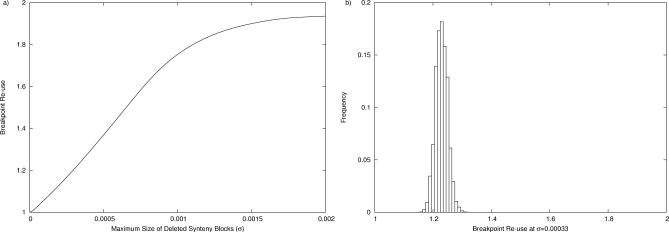
Breakpoint Re-Use Rate as a Function of *σ,* the Maximal Size of Deleted Synteny Blocks, and Its Distribution at *σ =* 0.00033 (A) Breakpoint re-use rate as a function of the maximal size of deleted synteny blocks (as the proportion of the whole genome length). Deletion of blocks shorter than 1 Mb as in [4] (assuming that the human genome is ≈3,000 Mb long, *σ* = 1 Mb/3,000 Mb ≈ 0.00033) results in low breakpoint re-use (≈1.2). The plot shows simulations for a genome with 25,000 genes and 320 reversals (in this case, *σ =* 0.00033 corresponds to deleting all synteny blocks shorter than nine genes). (B) The distribution of breakpoint re-use at *σ* = 0.00033 with a mean of 1.23 and a standard deviation of 0.02 (100,000 simulations). The maximum breakpoint re-use rate in this simulation was 1.33, and it appeared only once.

Figure 7 describes a simulation similar to the Sankoff-Trinh simulations, but in *σ* rather that in *θ* coordinates (all synteny blocks shorter than *σ* × *GenomeLength* are deleted). It is as good as Figure 5 for refuting FBM since the breakpoint re-use eventually increases when *σ* increases. However, one can see that breakpoint re-use is low at *σ* = 0.00033 (corresponding to 1 Mb, the maximal size of deleted blocks in [4]) and it is nowhere close to the observed human–mouse breakpoint re-use for any realistic values of parameter *σ*. In 100,000 simulations, the breakpoint re-use never reached the value 1.37 specified in Corollary 5, indicating that reaching such high breakpoint re-use is highly unlikely in the RBM framework.

We admit that since the choice of 1 Mb (*σ* = 0.00033) as the threshold for the deleting short synteny blocks is somewhat arbitrary, one can argue that the breakpoint re-use becomes large when *σ* exceeds 0.00150 (≈5 Mb). Therefore, one can argue that if Pevzner and Tesler [4] had chosen 5 Mb as the threshold for synteny block deletion, they would fall into the trap described in [7]. Below we explain a flaw with this counter-argument.

Indeed, in this case all synteny blocks shorter than 5 Mb would have to be deleted, and thus would have to be declared to be the breakpoint regions rather than the synteny blocks (for *σ* = 0.00150). It would result in a genome with an extremely high proportion of breakpoint regions (as opposed to 5% reported in [4] for 1 Mb threshold). Application of scan statistics to such a genome would not reveal any surprising breakpoint clustering, and the conclusion that evolution follows RBM would be confirmed—therefore, in this case [4] would never be written (let alone, published). This flawed “counter-argument” illustrates the key problem with [7]: it never took into account or even commented on the 5%–95% split between breakpoint regions and synteny blocks in the human and mouse genomes, the key argument against RBM. The rebuttal of RBM is based on *both* arguments (breakpoint-re-use and 5%–95% split) and [4] never claimed that breakpoint re-use *alone* invalidates RBM. Therefore, the rebuttal of [4] based solely on the breakpoint re-use argument (as in [7]) is flawed.

We emphasize that Figures 4 and 6 represent rather similar simulations and differ mainly in the choice of parameters for representing the results of these simulations (*θ* versus *σ*). There is no intrinsic advantage in choosing one simulation over another; the only difference is that one of these simulations (*θ*) is difficult to connect to the realities of the human–mouse analysis, while the other one (*σ*) has a clear interpretation. We also remark that for typical parameters, the Sankoff-Trinh “gene deletion” process is not dramatically different from the Pevzner-Tesler “synteny block deletion” process. For example, even if half of all genes are deleted (*θ* = 0.5), the Sankoff-Trinh simulation deletes (on average) 1/2*^i^* blocks of size *i*; i.e., removes mainly short blocks as in [46]. Of course, there is no one-to-one correspondence between the Sankoff-Trinh and Pevzner-Tesler deletion processes: some blocks shorter than the threshold are retained, and some blocks larger than the threshold are deleted in the Sankoff-Trinh simulation.

To better compare the Sankoff-Trinh gene deletion process with the synteny block deletion process, one may switch to parameter *γ*, the proportion of the total size of deleted blocks (this parameter can be directly computed for the Sankoff-Trinh simulation). For *γ* = 0.05 corresponding to the 5% proportion of the breakpoint regions in human–mouse comparison, the breakpoint re-use is small (≈1.2). The fact that it becomes as large as 1.9 for *γ* = 0.3 is irrelevant, since it does not reflect the reality of human–mouse comparison: indeed, we do not find that 30% of the human genome is formed by breakpoint regions that do not exhibit similarity with other mammalian genomes and have few orthologous genes. Again, if the human–mouse analysis in [4] revealed that the breakpoint regions account for a third of the genome, the paper [4] would never be written.

## Discussion

Nadeau and Taylor [18] proposed RBM based on a single observation: the exponential distribution of human–mouse synteny block sizes. There is no doubt that jumping to this conclusion was not fully justified mathematically: there are many other models (e.g., FBM) that lead to the same exponential distribution of the sizes of the “visible” synteny block. Apart from the 20-year old legacy, human and mouse genomes provide no evidence that would allow one to claim that RBM is correct and FBM is not; indeed, all statistical support for RBM immediately translates into statistical support for FBM. From this perspective, it is not clear how one can favor RBM over FBM without a single piece of evidence that holds for RBM but is violated for FBM. Pevzner and Tesler [4] presented the first evidence that RBM is in conflict with mammalian genomic architectures. Sankoff and Trinh [6,7] argued that the Pevzner-Tesler arguments against RBM are flawed. We acknowledge the important contribution of [6] in raising awareness that there are many subtle details and parameters in rearrangement analysis. At the same time, we emphasize that [6] did not present any arguments against FBM and did not connect their simulations with the realities of mammalian genomes.

Perusal of the UCSC Genome Browser (http://genome.ucsc.edu) reveals large numbers of short adjacent regions corresponding to parts of several chromosomes [49]. For example, the antibody regions in mammalian genomes show signs of multiple recurrent rearrangements. However, until recently, it remained unclear whether these regions reflect genome rearrangements (relevant to this paper), or duplications/assembly errors/alignment artifacts [50]. While previous studies attributed the fragile regions to high repeat density, high recombination rate, or pairs of tRNA genes, it remained unclear how to distinguish “true” short synteny blocks from computational artifacts [50].

**Figure 8:**
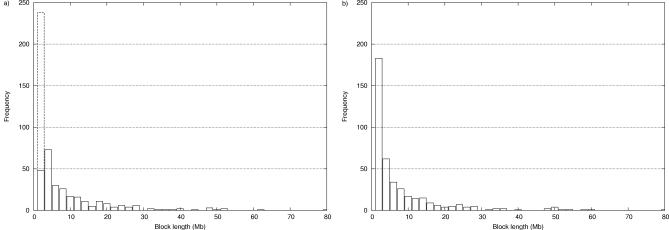
Distribution of the Synteny Block Sizes between the Human and Mouse Genomes Distribution of the synteny block sizes between the human and mouse genomes based on (A) 281 synteny blocks from [46] with extra 190 “hidden” short synteny blocks as predicted in [4] (this figure corresponds to Figure 1, center panel in [4]); and (B) 566 human–mouse synteny blocks derived from 1,338 multispecies conserved segments in [22]. The large number of confirmed short synteny blocks (leftmost bar in [B]) is already in conflict with the exponential distribution imposed by RBM. Moreover, the leftmost bar in (B) represents only the currently known short synteny blocks and does not even account for still unknown “hidden” synteny blocks that may have evaded the computational techniques in [22].

When RBM was formalized in 1984 [18], the short blocks in the human–mouse comparison were not available. By 2003, many short blocks were found, but it was not possible to decide which of them (if any) were real synteny blocks and which represented algorithmic or statistical artifacts. Acknowledging that these newly found short blocks were unreliable, Pevzner and Tesler [4] did not use any of them to refute RBM. Instead, they proved that such short blocks exist (without finding them) and predicted that the distribution of the synteny block sizes looks like Figure 8A (with an abnormally high bar corresponding to “hidden” short blocks). Recently, Ma et al. [22] finally revealed some short synteny blocks via the analysis of multiple mammalian genomes. Their distribution (Figure 8B) is remarkably similar to the distribution predicted by Pevzner and Tesler in 2003 [4] (Figure 8A).

The paper [4] has been cited in many biological papers, and we feel it is important to resolve the controversy that now confuses many researchers studying genome evolution. Since the rebuttal of RBM is based on a sophisticated theorem for computing rearrangement distances, few biologists can grasp all the details of both [4] and [6]. Fortunately, since both [4] and [6] use only computational arguments and simulations to refute/support RBM, this controversy (different from some biological controversies) is easy to resolve: one should simply check all computational arguments and simulations. In this paper, we developed algorithms for analyzing 3-breaks that generalize the standard rearrangements and make the analysis of rearrangements more transparent. We further analyzed the effects of transpositions (and other 3-breaks) on breakpoint re-use and came to the conclusion that even if transpositions and three-way fissions/fusions were dominant rearrangement operations, the arguments against RBM still hold. While one can still argue that rearrangements even more complex than 3-breaks (e.g., 4-breaks) are common, this argument is not supported by existing biological knowledge. We also reproduced the simulations from [6] and came to the conclusion that the “block deletion” argument in [7] is flawed, similarly to the already refuted “synteny blocks” argument in [6].

If RBM is put to rest in favor of FBM, one has to answer the question of what makes certain regions break and others not break. Peng et al. [20] argued that long regulatory regions and inhomogeneity of gene distribution in mammalian genomes might be responsible for the breakpoint reuse phenomenon. The link between rearrangements and regulatory regions was explored in depth by Kikuta et al. [16], who argued that long-range interactions between genes and their regulatory regions might explain solid and fragile regions in the genomes. However, revealing all factors responsible for genomic fragility and discovery of all fragile regions in the human genome remains an open problem.

## Methods

### 

A computational approach based on comparison of gene orders was pioneered by David Sankoff [51,52]. Since some methods and notations used in this paper differ from the previous papers, below we briefly review the key concepts/methods that are relevant for this paper and put them in the context of previous studies.

Initially, genome rearrangements were modeled by a combinatorial problem of sorting by reversals, as described below. The order of genes in two organisms is represented by permutations *π = π_1_π_2_…π_n_* and *σ* = *σ_1_σ_2_…σ_n_*. A *reversal ρ*(*i,j*) of an interval [*i,j*] is the permutation

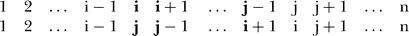



The reversal *ρ*(*i,j*) has the effect of reversing the order of *π_i_π_ι+1_…π_j_* and transforming *π_1_…π_i-1_π_i_…π_j_π_j+1_…π_n_* into *π⋅ρ*(*i,j*) = *π_1_…π_i-1_π_j_…π_i_π_j+1_…π_n_*. Given permutations *π* and *σ*, the reversal distance problem is to find a series of reversals *ρ_1_*,*ρ_2_*,*…*,*ρ_t_* such that *π·ρ_1_·ρ_2 ...·_ρ_t_ = σ* and *t* is minimal. We call *t* the reversal distance between *π* and *σ*. Sorting *π* by reversals is the problem of finding the reversal distance *d*(π) between *π* and the identity permutation (12...*n*).

We extend a permutation *π = π_1_π_2_…π_n_* by adding *π_0_* = 0 and *π_n+1_* = *n* + 1. We call a pair of elements (*π_i_,π_i+1_*), 0 ≤ *i* ≤ *n*, of *π* an *adjacency* if *|π_i_ - π_i+1_|* =1, and a *breakpoint* if *|π_i_* − *π_i+1_|* > 1. It is easy to see that *d*(*π*) *≥ b*(*π*) / 2, where *b*(*π*) is the number of breakpoints in *π*. However, the estimate of reversal distance in terms of breakpoints is very inaccurate. Bafna and Pevzner [53] showed that another parameter (size of a maximum cycle decomposition of the breakpoint graph) estimates reversal distance with much greater accuracy.

Originally, the *breakpoint graph* of a permutation *π* was defined as an edge-colored graph *G*(*π*) with *n* + 2 vertices {*π_0_, π_1_, …, π_n,_ π_n+1_*} = {0, 1, ..., *n* + 1}. We join vertices *π_i_* and *π_i+1_* by a *black* edge for *0* ≤ *i* ≤ *n*. We join vertices *π*_*i*_ and *π*_*j*_ by a *gray* edge if *π*_*i*_ − *π*_*j*_ = 1. A *cycle* in an edge-colored graph *G* is called *alternating* if the colors of every two consecutive edges of this cycle are distinct. It is easy to see that *G*(*π*) contains an alternating Eulerian cycle. Therefore, there exists a *cycle decomposition* of *G*(*π*) into edge-disjoint alternating cycles (every edge in the graph belongs to exactly one cycle in the decomposition). We are interested in the decomposition of the breakpoint graph into a *maximum* number *c*(*π*) of edge-disjoint alternating cycles.

Cycle decompositions play an important role in estimating reversal distance. Bafna and Pevzner [53] proved the bound *d*(*π*) *≥ n* + 1 − *c(π)*, which is much tighter than the bound in terms of breakpoints *d*(*π*) *≥ b*(*π*) / 2.

Finding a maximal cycle decomposition is a difficult problem. Fortunately, in the more biologically relevant case of *signed permutations,* this problem is trivial. Genes are *directed* fragments of DNA, and a sequence of *n* genes in a genome is represented by a *signed* permutation on {*1,...,n*} with a “+” or “−” sign associated with every element of *π*. In the signed case, every reversal of fragment [*i,j*] changes both the order and the signs of the elements within that fragment. We are interested in the minimum number of reversals *d*(*π*) required to transform a signed permutation *π* into the identity signed permutation (+1+2...+*n*).

The concept of a breakpoint graph extends naturally to signed permutations by mimicking every directed element by two undirected elements, which substitute for the tail and the head of the directed element [53]. For signed permutations, the bound *d*(*π*) *≥ n* +1 − *c*(*π*) approximates the reversal distance extremely well. Hannenhalli and Pevzner [54] showed that

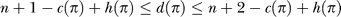
where *h*(*π*) is the number of hurdles in *π*.

In the model of the multichromosomal genomes we consider, every gene is represented by an integer whose sign (“+” or “–”) reflects the direction of the gene. A chromosome is defined as a sequence of genes, while a genome is defined as a set of chromosomes. Given two genomes *π* and *Γ*, we are interested in a most parsimonious scenario of *evolution* of *Π* into *Γ* (i.e., the shortest sequence of rearrangement events [defined below] required to transform *Π* into *Γ*). We assume that *Π* and *Γ* contain the same set of genes.

Let *Π* be a multichromosomal genome. Every chromosome *π* in *Π* can be viewed either from left to right (i.e., as *π* = (*π_1_…π_n_*)) or from right to left (i.e., as −*π* = (*−π_n_…*−*π_1_*)), leading to two equivalent representations of the same chromosome (i.e., the *directions* of chromosomes are irrelevant). The four most common elementary rearrangement events in multichromosomal genomes are reversals, translocations, fusions, and fissions, defined below.

Let *π* = *π_1_…π_n_* be a chromosome and 1 ≤ *i* ≤ *j* ≤ *n*. A *reversal ρ*(*π,i,j*) on a chromosome *π* rearranges the genes *inside π* = *π_1_…π_i-1_π_ι_…π_j_π_j+1_…π_n_* and transforms *π* into *π_1_…π_i-1_* − *π_j_…*−*π_i_π_j+1_…π_n_*. Let *π* = *π_1_…π_n_* and *σ* = *σ_ι_…σ_m_* be two chromosomes and 1 ≤ *i* ≤ *n* +1, 1 ≤ *j* ≤ *m* + 1. A translocation *ρ*(*π,σ,i,j*) exchanges genes *between* chromosomes *π* and *σ* and transforms them into chromosomes *π_1_…π_i-1_σ_j_…σ_m_* and *σ_ι_…σ_j-1_π_i_…π_n_* with (*i* – 1) + (*m* – *j* + 1) and (*j* – 1) + (*n* – *i* + 1) genes, respectively. We denote as *Π·ρ* the genome obtained from *Π* as a result of a rearrangement (reversal or translocation) *ρ*. Given genomes *Π* and *Γ*, the genomic sorting problem is to find a series of reversals and translocations *ρ_1_*,*ρ_2_*,*…*,*ρ_t_* such that *Π·ρ_1_·ρ_2_·…·ρ_t_* = *Γ* and *t* is minimal. We call *t* the genomic distance between *Π* and *Γ.* The genomic distance problem is the problem of finding the genomic distance *d*(*Π,Γ*) between *Π* and *Γ.*

A translocation *ρ*(*π,σ,n +* 1,1) concatenates the chromosomes *π* and *σ*, resulting in a chromosome *π_1_…π_n_σ_*1*_…σ_m_* and an *empty* chromosome *Ø*. This special translocation, leading to a reduction in the number of (nonempty) chromosomes, is known in molecular biology as a *fusion*. The translocation *ρ*(*π,Ø,i,1*) for 1 < *i* < *n* “breaks” a chromosome *π* into two chromosomes: (*π_1_…π_i-1_*) and (*π_i_…π_n_*). This translocation, leading to an increase in the number of (nonempty) chromosomes, is known as a *fission.*

## Supporting Information

Text S1 An Overview of RBM and FBM (123 KB PDF)

## Abbreviations

FBM: fragile breakage model
RBM: random breakage model
