# Supplementary material for: Are There Rearrangement Hotspots in the Human Genome?
Source: PLoS Comput Biol. 2007 Nov 9;3(11):e209. doi: 10.1371/journal.pcbi.0030209 (PMC2065889; doi:10.1371/journal.pcbi.0030209)
Supplement: Supplementary file 1 [file pcbi.0030209.sd001.doc]

# Appendix. An overview of RBM and FBM

In this appendix we briefly review RBM and FBM models of chromosomal evolution (see [1, 2Error: Reference source not found]).

The Random Breakage Model (RBM) assumes a random (i.e., uniform and independent) distribution of chromosome rearrangement breakpoints and is supported by the observation that the lengths of synteny blocks shared by human and mouse are well fitted by the predicted distribution imposed by the random breakage model.

The theoretical distribution of lengths of conserved segments is an exponential distribution with density function

*f(x) = 1/L·e^-x/L^*

where *L* is the average length of all segments. Nadeau and Taylor [1] were able to estimate *L* (and therefore the number of still undiscovered segments) from the small set of already discovered segments. The relatively small departure from an exponential distribution was attributed to missing information about some conserved segments.

Pevzner and Tesler [2Error: Reference source not found] identified 281 synteny blocks shared by human and mouse of size at least 1 Mb. Although the number of synteny blocks is higher than the Nadeau–Taylor predictions, the lengths of the blocks still fit the exponential distribution (Fig. 1 Left in [2Error: Reference source not found]), another argument in favor of the random breakage model. However, a different type of evidence derived from genome rearrangements studies reveals that there exists an unexpectedly large number of closely located breakpoints that cannot be explained by the random breakage model. This analysis implies that in addition to the segments shown in Fig. 1 Left in [2Error: Reference source not found], another 190 “short” synteny blocks, typically <1 Mb in length, exist. These blocks were never discovered in the comparative mapping studies, and moreover, most of them are hard to find even with available human and mouse sequences. If the breakpoints are located very close to each other (e.g., within a few nucleotides or even at the same position), the corresponding very short blocks may be undetectable by alignment analysis. Moreover, some short blocks may be deleted in the course of evolution. However, the rearrangement analysis confirms the existence of such breakpoints, even in the absence of statistically significant sequence alignments. The existence of these short blocks immediately implies that an exponential distribution is not a good fit to reality, thus pointing to limitations of the random breakage model (Fig. 1 Center in [Error: Reference source not found2]). In other words, the rearrangement analysis of human and mouse genomes reveals clumps of closely located breakpoints that cannot be explained by the random breakage model.

The surprisingly large number of breakpoint clumps is an argument in favor of a different model of chromosome evolution, called the Fragile Breakage Model. This model postulates that the breakpoints occur mainly within relatively short fragile regions (hot spots of rearrangements). If one assumes that the fragile regions are uniformly distributed through the genome then the fragile and random breakage models lead to identical estimates for the number of long segments. In some sense, the random breakage model can be viewed as an excellent null hypothesis for a certain level of resolution and genome heterogeneities. However, the RBM and FBM generate very different predictions when it comes to short segments that were below the granularity level of comparative mapping studies. We emphasize that by reusing breakpoints we do not mean multiple use of exactly the same genomic position as an endpoint of rearrangements, but rather the fact that the breakpoint regions host endpoints for multiple rearrangement events.

### FBM versus RBM

If positions of *n* breakpoints in the genome are given by random variables u_i_ in [0,1], the segment sizes are *y_i_ = u_i_ - u_i-1_*_._ Pevzner and Tesler [Error: Reference source not found2] ignore the chromosome endpoints, assume *n = N_b_ – 1 = 280* and, similarly to Nadeau and Taylor [1], do not consider short blocks (<1 Mb). To test the random breakage model, they follow the approach described in Churchill et al. [3] and use the Kolmogorov–Smirnov test, which measures the largest difference between the empirical and theoretical distribution functions:

*D_n_ = max{ max_1≤i≤n_(i/n – u_i_), max_1≤i≤n_(u_i_ - (i-1)/n) }*

The computed Kolmogorov–Smirnov statistic is *D_280_=0.085*, which comes close to the estimate of *0.095* computed by Sankoff et al. [4] based on comparative mapping data for 1,423 genes and only 130 blocks. The probability (*P* value) that the Kolmogorov–Smirnov statistic *D_280_* is *>0.085* for the uniform distribution is *0.032*.

Ideally, these inferences should be based on complete data about segment lengths. However, the information about short segments may be hard to obtain even with available draft human and mouse sequences. Churchill et al. [3] model the missing data by assuming that if two breakpoint sites are within *locking distance* *a* then the conserved segment remains undetected. However, even this more flexible model is unable to explain the very large number *N_r_ = 190* of short unobserved segments.

In summary, the RBM is unable to explain a large number of short synteny blocks found by genome rearrangement analysis. At the same time, the truncated exponential density function *1/L e^x-aL^* fits the experimental data for synteny blocks longer than *a =* 1 Mb well. The FBM explains the fit between the distribution of long synteny blocks and the truncated exponential density function observed by Nadeau and Taylor [1], and a large number of short blocks that the Nadeau–Taylor statistics failed to explain.

# **References**

[1] Nadeau JH, Taylor BA (1984) Lengths of chromosomal segments conserved since divergence of man and mouse. Proceedings of the National Academy of Sciences 81: 814–818.

[2] Pevzner PA, Tesler G (2003) Human and mouse genomic sequences reveal extensive breakpoint reuse in mammalian evolution. Proceedings of the National Academy of Sciences 100: 7672–7677.

[3] Churchill GA, Daniels DL, Waterman MS (1990) The distribution of restriction enzyme sites in Escherichia coli. Nucleic Acids Research 18: 589–597. 10.1093/nar/18.3.589.

[4] Sankoff D, Parent MN, Marchand I, Ferretti V (1997) On the Nadeau-Taylor theory of conserved chromosome segments. Lecture Notes in Computer Science 1264: 262–274. 10.1007/3-540-63220-4_64.
